# Supplementary material for: A Critical View of the Application of the APEX Software (Aqueous Photochemistry of Environmentally-Occurring Xenobiotics) to Predict Photoreaction Kinetics in Surface Freshwaters
Source: Molecules. 2019 Dec 18;25(1):9. doi: 10.3390/molecules25010009 (PMC7017383; doi:10.3390/molecules25010009)
Supplement: Supplementary file 1 [file molecules-25-00009-s001.zip › 3.2.4_gcc-4.4.0.zip!/3.2.4_gcc-4.4.0/3.2.4_gcc-4.4.0/doc/octave/html/interpreter/Call-Stack.html]

Call Stack - Untitled


Previous: Debug Mode,
Up: Debugging


---

### 13.5 Call Stack

— Loadable Function: [stack, idx] **dbstack** (n)  
> Print or return current stack information. With optional argument
> n, omit the n innermost stack frames.
>
>
>
> **See also:** dbclear, dbstatus, dbstop.

— Loadable Function: **dbup** (n)  
> In debugging mode, move up the execution stack n frames.
> If n is omitted, move up one frame.
>
>
>
> **See also:** dbstack.

— Loadable Function: **dbdown** (n)  
> In debugging mode, move down the execution stack n frames.
> If n is omitted, move down one frame.
>
>
>
> **See also:** dbstack.
